# Intracranial inoculation rapidly induces Nipah virus encephalitis in Syrian hamsters

**DOI:** 10.1371/journal.pntd.0012635

**Published:** 2024-10-28

**Authors:** Manmeet Singh, Kerry Goldin, Meaghan Flagg, Brandi N. Williamson, Tessa Lutterman, Brian Smith, Emmie de Wit

**Affiliations:** 1 Laboratory of Virology, Division of Intramural Research, National Institute of Allergy and Infectious Diseases, National Institutes of Health, Hamilton, Montana, United States of America; 2 Rocky Mountain Veterinary Branch, Division of Intramural Research, National Institute of Allergy and Infectious Diseases, National Institutes of Health, Hamilton, Montana, United States of America; The University of the West Indies, JAMAICA

## Abstract

Nipah virus (NiV) is a highly pathogenic Paramyxovirus associated with outbreaks in Malaysia, Bangladesh, and India with high mortality rates. NiV infection causes fatal respiratory and neurological disease. The majority of survivors suffer from long-term neurological sequelae or late onset and relapsed encephalitis. The pathogenesis of neurological disease is complex and has not been able to be studied in current animal models as they are skewed towards the development of lethal respiratory disease rather than neurological disease. Although NiV neurological disease can be observed in animal models, there is currently no model where the majority of animals consistently develop neurological disease. Here, we developed a new Syrian hamster (*Mesocricetus auratus*) model to mimic neurological disease in humans. Hamsters were inoculated intracranially in the cerebellomedullary cistern with different doses of NiV, strain Malaysia. Intracranial NiV inoculation in the cerebellomedullary cistern resulted in a rapid progression towards severe neurological disease requiring euthanasia. High Nipah viral loads were detected in the brains, and NiV spread from the CNS to the lungs. Histopathologic examination of the brain showed ischemic necrosis, often accompanied by marked edema and hemorrhage. NiV antigen was detected primarily in meninges and cerebellum, but rarely observed in brain parenchyma. These histological lesions were different from the typical lesions observed in NiV-infected humans. Thus, despite the consistent development of neurological disease, intracranial inoculation does not result in a model representative of NiV neurological disease.

## Author summary

Nipah virus causes outbreaks of severe respiratory and neurological disease in Bangladesh and India. The majority of Nipah virus-infected patients succumb to the disease; survivors often suffer from lasting neurological consequences of infection. Although Nipah virus infection of the brain is thus an important component of Nipah virus disease, most studies to date have focused on the severe respiratory disease also caused by Nipah virus. In an attempt to develop a robust model to study Nipah virus neurological disease, we infected Syrian hamsters with Nipah virus by injecting the virus directly into the cerebellomedullary cistern in the brain. Animals quickly developed severe neurological disease requiring euthanasia and the virus could be detected at high levels in the brain, and lower levels in the lungs. Interestingly, the changes observed in the brains of infected animals were not the same as those observed in Syrian hamsters infected via other routes, nor those observed in human cases of Nipah virus infection in Malaysia, indicating that the inoculation route determines the course of disease in the brain.

## Introduction

Nipah virus (NiV) is a Paramyxovirus belonging to the Henipavirus genus. NiV infection is associated with acute respiratory illness and encephalitis in infected individuals, with a high case fatality rate [1,2]. The majority of NiV-infected patients develop neurological disease [2–4] and survivors may develop long lasting neurological complications [5]. During acute encephalitis, multifocal discrete high-intensity lesions throughout the brain were seen in Magnetic Resonance Imaging [6]. A small proportion of survivors from the NiV outbreak in Malaysia experienced relapse or late-onset encephalitis months and even years after the initial infection [7–9]. Encephalitis in central nervous system (CNS) specimens from patients who succumbed to NiV infection in Malaysia was characterized by vasculitis accompanied by thrombosis, perivascular cuffing, and necrosis [10]. Clinical data and specimens from human cases of NiV infection are limited. Thus, animal models play an important part in understanding NiV pathogenesis. NiV has a broad species tropism, and several animal models of infection including pigs, African green monkeys, Syrian hamsters, and ferrets have been developed [11]. However, the primary disease manifestation observed in the current animal models is severe, lethal respiratory disease, while neurological signs are most often mild or absent. NiV neurological disease can be observed in hamsters, e.g. when they are inoculated with a relatively low dose; however, animals do not consistently display neurological signs even in those models [12–14]. Thus, there is a necessity to develop an animal model where the majority of NiV inoculated animals develop overt neurological disease. Such a model would also allow the efficacy testing of potential antivirals when administered once virus may have already reached the CNS. Here, we developed a novel model to study NiV infection in the CNS of Syrian hamsters. We directly targeted the CNS of hamsters through intracranial inoculation with NiV in the cerebellomedullary cistern. We found that intracranial inoculation in the cerebellomedullary cistern induces a rapidly lethal neurological disease with 100% of the animals showing neurological signs such as seizures, head tilt, paralysis, and ataxia.

## Methods

### Ethics statement

Approval of animal experiments was obtained from the Institutional Animal Care and Use Committee of the Rocky Mountain Laboratories, National Institutes of Health. Experiments are carried out in an Association for Assessment and Accreditation of Laboratory Animal Care (AAALAC) International–accredited facility, according to the institution’s guidelines for animal use, following the guidelines and basic principles in the NIH Guide for the Care and Use of Laboratory Animals, the Animal Welfare Act, U.S. Department of Agriculture, and the U.S. Public Health Service Policy on Humane Care and Use of Laboratory Animals. Work with infectious Nipah virus under BSL4 conditions was approved by the Institutional Biosafety Committee (IBC). Inactivation and removal of samples from high containment was performed per IBC-approved standard operating procedures.

### Virus and cells

Nipah virus strain Malaysia/199901924 (GenBank AF212302; cell culture passage 2) was provided by the Special Pathogens Branch of the Centers for Disease Control and Prevention, Atlanta, GA, and propagated once in Vero E6 cells in DMEM supplemented with 2% fetal bovine serum (FBS), 1 mM L-glutamine, 50 U/ml penicillin and 50 μg/ml streptomycin; the resulting virus titer of this stock was 7.9x10^7^ TCID50/ml. NGS was performed to confirm the sequence of the virus was identical to that in GenBank. VeroE6 cells were maintained in DMEM supplemented with 10% fetal calf serum, 1 mM L-glutamine, 50 U/ml penicillin and 50 μg/ml streptomycin. Cells are tested monthly for the presence of mycoplasma and have remained negative.

### Animal experiment

Nine groups of six, 4–6 week-old female Syrian hamsters (Envigo laboratories) were inoculated intracranially with different inoculation doses of NiV-M ranging from 0.008–3125 TCID50 in a total volume of 30μl per animal. A group of mock animals was inoculated with 30μl DMEM. Animals were randomly assigned to groups by veterinary care staff. All the inocula were backtitered to confirm the dose of inoculation and were within range. Inocula were administered to anesthetized animals intracranially via the cerebellomedullary cistern, chosen for its technical feasibility and personnel safety in BSL4. The procedure was as follows: head and neck were shaved and wiped with 70% ethanol to palpate the foramen magnum [15]. The animal was placed on a custom-made anesthesia table with the animal’s head secured in such a way that the nose and mouth of the animal were on the custom-made surgical table, connected to an inhaled anesthesia gas breathing tube. The eyes were lubricated with an ophthalmic solution before performing injections. The animal’s head was restrained with a forceps and positioned with the neck bent (90–120 degrees). A 30G needle was inserted into the skin to a depth of 4 mm at the back of the foramen magnum and the inoculum was slowly injected into the cerebellomedullary cistern. Prior to inoculation with Nipah virus, pilot experiments were performed including injections with evans blue or saline, to determine the needle depth required for inoculation into the cerebellomedullary cistern. Some animals (<10%) needed to be euthanized due to post-operational side effects from the inoculation procedure and were not included in survival curve. All animals were monitored for end point-criteria post inoculation and survived animals were euthanized at 14dpi. The predetermined endpoint criteria for this experiment included signs of circling, head tilt, ataxia, lameness, paralysis, seizure, weight loss >15% and labored breathing. At necropsy, tissue samples were collected from the cortical hemisphere of the brain and from the lungs.

For the intraperitoneal infection route, 6800 TCID50 (100 LD50) of NiV-M in a total volume of 500μl was injected into the intraperitoneal cavity of 6–8 week-old hamsters (Envigo laboratories). All animals were euthanized by 6 dpi due to signs of severe respiratory distress.

### RNA extraction and qRT-PCR

Total RNA was extracted from different tissues using the RNeasy Kit (Qiagen) following the manufacturer’s instructions. ≤30mg of tissue was used for RNA extraction. Five μl of RNA was used in a one-step real-time RT-PCR targeting the nucleoprotein (NP) gene [16] using the QuantiFast Probe Kit (QIAGEN) according to the manufacturer’s instructions. In each run, standards with known copy numbers were run in parallel to calculate copy number in the samples.

### 16S rRNA sequencing

RNA extracted from brain tissue was converted into cDNA using SuperScript III First-Strand Synthesis System (Invitrogen) and PCR amplified using iProof High-Fidelity DNA Polymerase (BioRad) with the following primers 5’-CCAGCAGCCGCGGTAATACG3’ and 5’-ATCGGYTACCTTGTTACGACTTC-3’. The amplified product was PCR purified using NucleoSpin Gel and PCR Clean-up kit (Macherey-Nagel) and submitted for Sanger sequencing.

### Virus titration

Virus titrations were performed by endpoint titration on Vero E6 cells. Cells were inoculated with 10-fold serial dilutions of tissue homogenates in DMEM. One hour after inoculation of cells, the cells were washed with PBS and 100 μl DMEM supplemented with 2% fetal bovine serum, 1 mM L-glutamine, penicillin (50 U/ml), and streptomycin (50 μg/ml) was added. The cytopathic effect was scored three days after inoculation, and the TCID50 per gram of tissue was calculated.

### Histopathology, immunohistochemistry (IHC) and in situ hybridization (ISH)

All tissues (brain and lungs) harvested at the time of necropsy were fixed in 10% neutral-buffered formalin for ≥7 days. Tissues were embedded in paraffin, sectioned to 5μm, and stained with H&E for evaluation of histological lesions. To detect NiV antigen by IHC, tissues were stained with a rabbit anti-nucleocapsid antibody (GenScript). Briefly, tissues were stained with a 1:500 dilution of antibody, followed by incubation with a secondary alkaline phosphatase linked antibody ImmPRESS VR horse anti-rabbit IgG polymer (Vector Laboratories) and counterstaining with hematoxylin. The immunohistochemistry (IHC) assay was carried out on a Discovery ULTRA automated-staining instrument (Ventana Medical Systems) with a Discovery ChromoMap DAB kit (Roche Tissue Diagnostics). A board-certified veterinary anatomic pathologist, blinded to group assignment of the animals, evaluated all tissue slides.

Detection of NiV viral RNA was performed on formalin fixed tissue using the RNAscope VS Universal AP assay (Advanced Cell Diagnostics Inc.) on the Ventana Discovery ULTRA stainer using an RNAscope 2.5 VS Probe- V-Nipah-sense consisting of 40 probe pairs targeting the positive sense RNA at base pairs 2400–4400 (Advanced Cell Diagnostics Inc. cat# 520079 according to the manufacturer’s instructions.

Histological lesions from brain sections stained with H&E were categorized into lymphoplasmacytic meningitis; encephalitis and lymphoplasmacytic with gliosis; malacia; vasculitis and fibrin thrombi; hemorrhage; and blood in ventricles/meninges. Each category was scored on the scale of 0 = none,1 = rare, 2 = mild, 3 = moderate and 4 = severe; scores per category were added up to reach a cumulative histology score.

Histological lesions from lung sections stained with H&E were categorized into lymphoid cuffing; lymphoplasmacytic interstitial pneumonia; presence of syncytial cells; interstitial and intra-alveolar macrophages; type II pneumocyte hyperplasia; perivascular and alveolar edema; vasculitis, and fibrin thrombi. Each category was scored on the scale of none = 0, rare = 1, 2 = mild, 3 = moderate and 4 = severe; scores per category were added up to reach a cumulative histology score. Immunohistochemistry scoring was as follows 0 = none/no positive cells; 1 = rare positive cells; 2 = few positive cells; 3 = moderate numbers of positive cells; 4 = abundant positive cells; scores per category were added up to reach a histology score (IHC).

### Cytokine assay

The 9-Plex Multiplex Luminex Assay (Hamsters cytokine Panel-1, SKU H101-KB) from Ampersand & Biosciences was used to test brain samples for the presence of nine cytokines (IL-2, IL-4, IL-10, IL-6, IFN-γ, MCP-1, MIP-1α, TNF-α, & VEGF) following the manufacturer’s instructions with the following changes: brain homogenates were prepared up to a 50mg/ml concentration in DMEM and irradiated with a dose of 8MRad to inactivate virus. The prepared tissue homogenate was used at 1:4 dilution in assay buffer. Plates were read using a Bioplex Luminex 200 instrument (BioRad), and data were analyzed using Bioplex (Biorad) and Excel (Microsoft) software. Observed concentration in pg/gm of tissue from NiV-M-infected samples compared to mock inoculated animals was calculated using the standard curve provided with the assay.

### ELISA

IgG antibody responses were measured in an ELISA using recombinantly expressed NiV-M glycoprotein G as described previously [16]. Recombinant G protein was produced and purified as described elsewhere [17]. Nunc Maxisorp plates (ThermoFisher) were coated with NiV G (50 ng in 100 μl per well, diluted in PBS) overnight at 4°C. Plates were blocked with 5% skim milk in PBS containing 0.05% Tween 20 (PBST) for 1.5 h at 4°C. After 3 washes with PBST, 100 μl of serum samples diluted in PBS (starting dilution 1:100) were added and the plates were incubated for 1 h at 37°C. Bound antibodies were detected after three washes with PBST using horseradish peroxidase (HRP)-conjugated anti-hamster IgG (Invitrogen). Following incubation for 1 h at 37°C, bound HRP was detected using the ABST Peroxidase substrate system (KPL). The absorbance was measured at 405 nm; sera were considered positive when absorbance was higher than three standard deviations above the mean of negative control sera.

### Statistical analysis

Statistical analysis was performed by the Log-rank (Mantel-Cox) test to compare survival curves. Statistical analysis was performed using Mann-Whitney test to compare differences in cytokine levels. SEM was calculated for all samples. P-values < 0.05 were considered statistically significant. All statistical calculations were done using GraphPad Prism version 10.1.

## Results

### Intracranial Nipah virus inoculation results in rapidly progressing neurological disease

**Fig 1:**
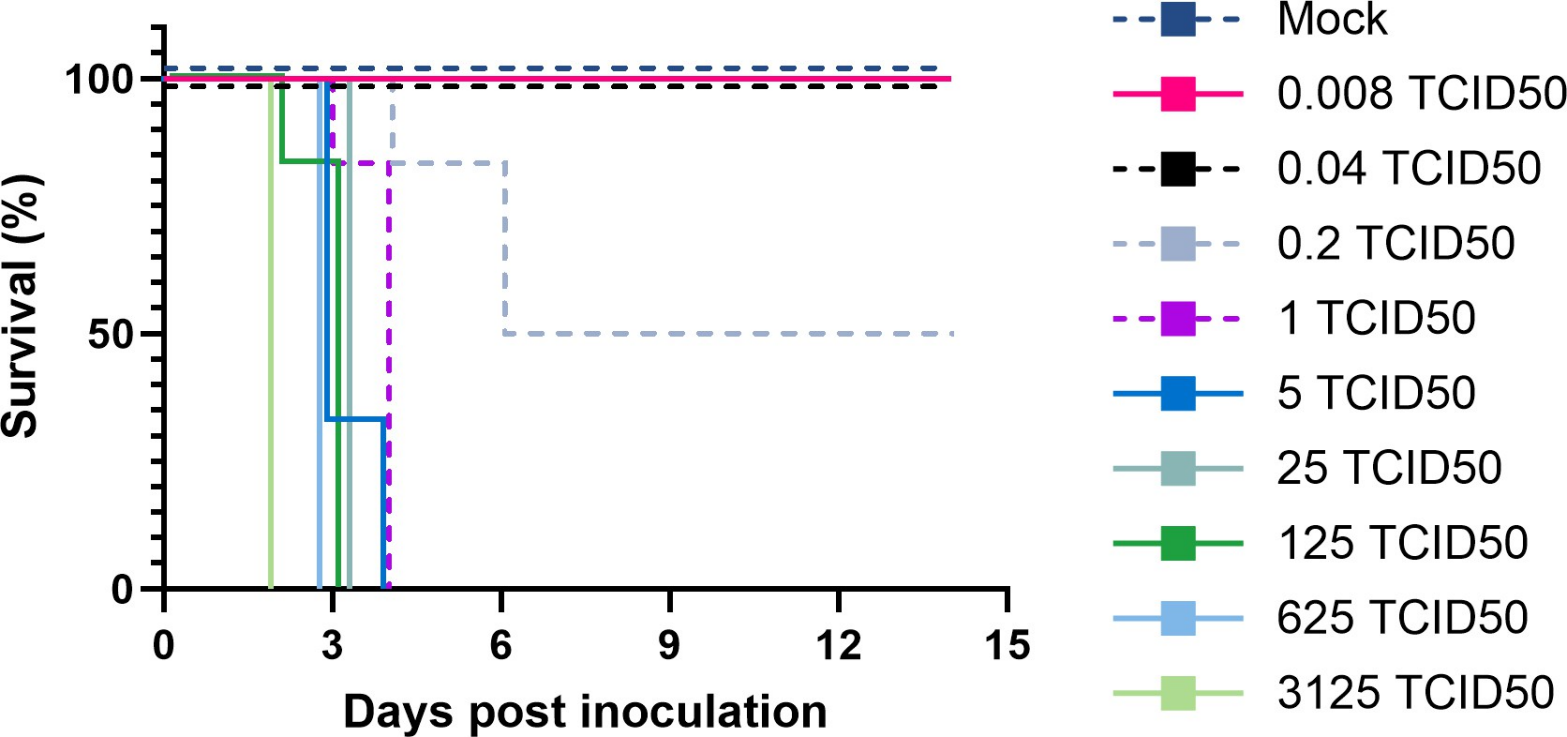
Intracranial inoculation of hamsters with NiV-M results in lethal disease. Groups of 6 hamsters were inoculated intracranially into the cerebellomedullary cistern with 0.008 to 3125 TCID50 NiV-M in 5-fold serial dilutions. The percentage of animals surviving over time is shown.

To determine the optimal dose of NiV that results in uniform neurological disease, nine groups of six hamsters were inoculated intracranially with 5-fold serial dilutions of NiV, Malaysia (NiV-M), ranging from 0.008 to 3125 TCID50. An additional group received the same volume of DMEM as a control inoculation. Animals inoculated with 1–3125 TCID50 NiV-M uniformly displayed severe neurological signs and reached endpoint criteria within 2–6 days post inoculation (dpi) (Fig 1). The time to euthanasia was dose-dependent, with the animals that received the highest dose reaching euthanasia criteria as early as 2 dpi, and animals that received lower doses reaching euthanasia criteria 1–2 days later. Of note, in the group inoculated with 625 TCID50, four out of six animals succumbed to the infection before they could be euthanized and samples collected. Lowering the inoculation dose to 0.2 TCID50 resulted in only 50% of animals requiring euthanasia between 4–6 dpi; no signs of disease were observed in the surviving animals. Doses of 0.04 TCID50 or below did not result in disease signs nor lethality. Animals requiring euthanasia primarily displayed neurological signs such as seizures, torticollis, paralysis, and ataxia. All mock-inoculated animals survived until study endpoint at 14 dpi (Fig 1).

### Nipah virus replicates to high titers in the brain

**Fig 2:**
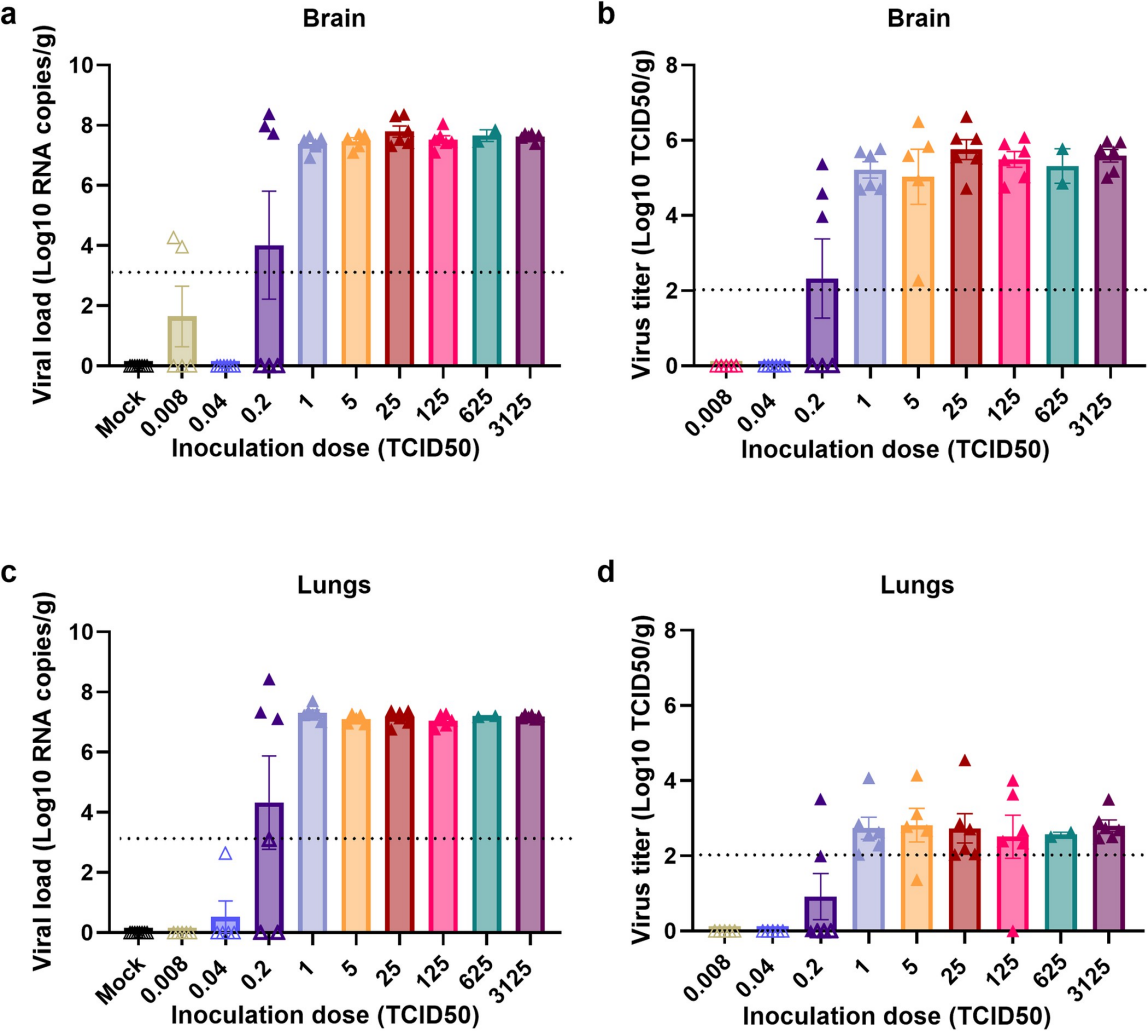
NiV-M disseminates outside of the CNS. Brain (a, b) and lungs (c, d) were collected at the time of euthanasia from hamsters inoculated intracranially with different doses of NiV-M and analyzed for the presence of viral RNA by qRT-PCR and for the presence of infectious virus. Open symbols represent animals that survived until the end of the experiment; closed symbols represent animals that reached euthanasia criteria. Bars represent geometric mean and error bars indicate standard error of the mean. Dotted lines represent lower limit of detection.

To assess whether different doses of inoculation led to varying levels of virus replication, brain and lung samples were collected at time of euthanasia and analyzed for the presence of viral RNA and infectious virus. The brains of animals that reached euthanasia criteria showed a consistently high viral load, regardless of the inoculation dose (Fig 2A). Similarly, high virus titers were observed in the brains of these animals (Fig 2B). Although viral RNA was detected in the brains of some surviving animals (Fig 2A), infectious virus could not be detected (Fig 2B).

To determine whether surviving animals were infected or not, we tested their sera collected at 14 dpi for the presence of anti-NiV glycoprotein G antibodies using ELISA. We did not detect antibodies against the NiV G glycoprotein in sera collected from surviving animals, including those where a low level of viral RNA was detected (S1 Fig), indicating that inoculation did not result in infection in the surviving animals.

### Nipah virus disseminates to the lungs upon intracranial inoculation

**Fig 3:**
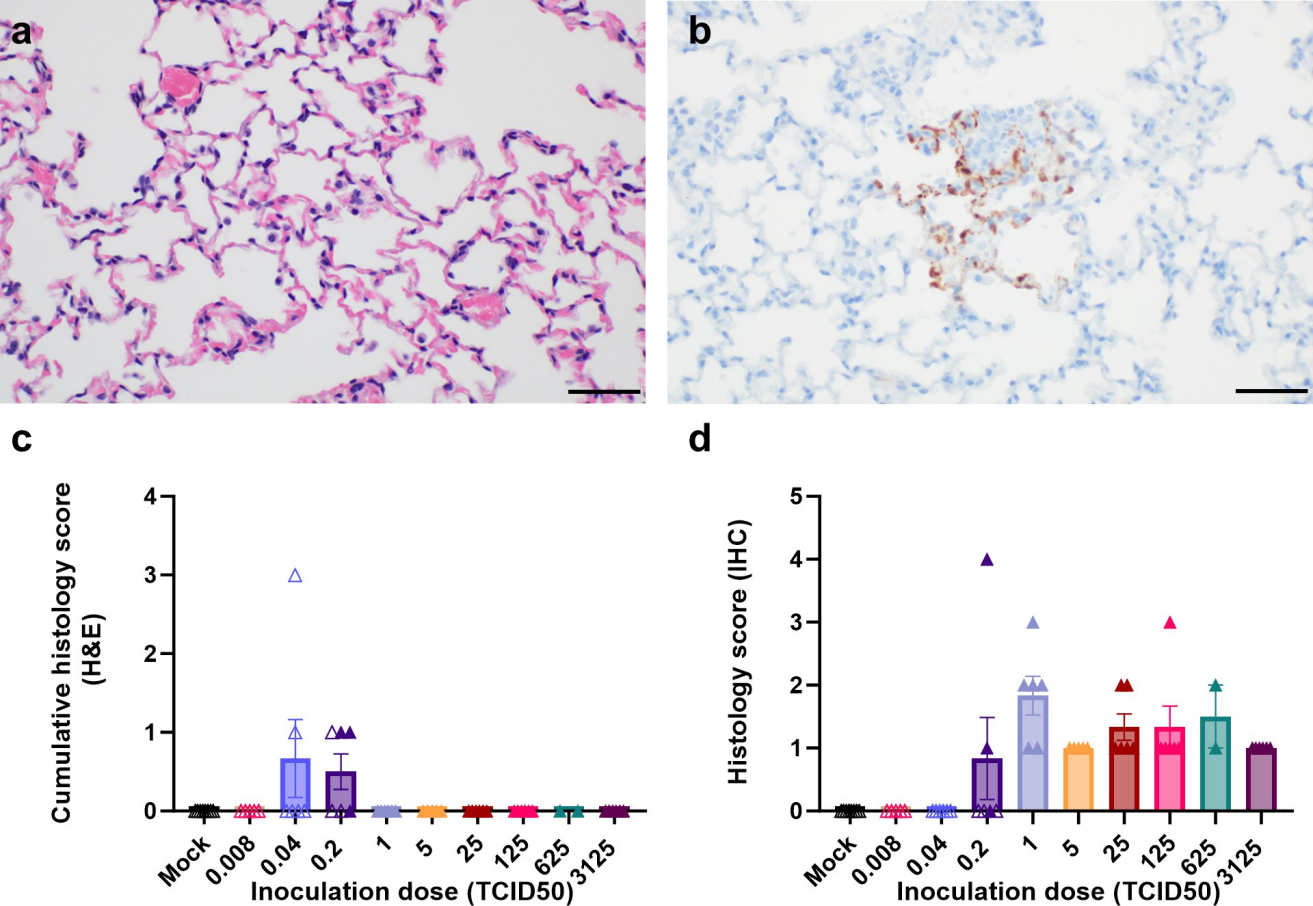
Absence of histological lesions in lungs of intracranially inoculated animals despite virus replication. At time of euthanasia, lungs were collected in formalin and sections were stained using hematoxylin and eosin (a) and immunohistochemistry using an anti-NiV N antibody was performed (b). Scale bar, 50μm. Presence of histological lesions and viral antigen were scored as described in Methods by a board-certified veterinary pathologist and graphed in (c) and (d), respectively. Open symbols represent animals that survived until the end of the experiment; closed symbols represent animals that reached euthanasia criteria. Bars represent mean and error bars indicate standard error of the mean.

Despite direct inoculation into the CNS, high viral loads were detected in the lungs of animals that reached euthanasia criteria (Fig 2C and 2D). However, virus titers in the lungs were lower than in the brains. Histological lesions were not observed in most of the lung sections collected at necropsy (Fig 3A and 3C); however, mild lung lesions were detected in animals inoculated with a low dose (0.2 TCID50) and euthanized 4–6 dpi. Based on the rapid development of disease signs requiring euthanasia, and the observation of mild lung lesions in animals that were euthanized at later timepoints after inoculation, it is likely that the typical NiV-associated pneumonia did not have time to develop.

In all animals that reached endpoint criteria, small foci of viral antigen were detected in type I and type II pneumocytes, and occasionally within endothelial cells (Fig 3B and 3D). These regions of positivity did not extend beyond one or two alveolar sacs. Together, these data suggest that NiV can disseminate out of the CNS after intracranial inoculation.

### Limited cytokine response in the brains of intracranially inoculated hamsters

The brains of animals were analyzed for the presence of nine different cytokines and chemokines. We compared tissue cytokine and chemokine levels between animals inoculated with 25 TCID50 that reached endpoint criteria to mock-inoculated animals euthanized at 14 dpi. The group inoculated with 25 TCID50 NiV-M was chosen for this analysis because all animals in this group reached endpoint criteria on the same day (3 dpi).

**Fig 4:**
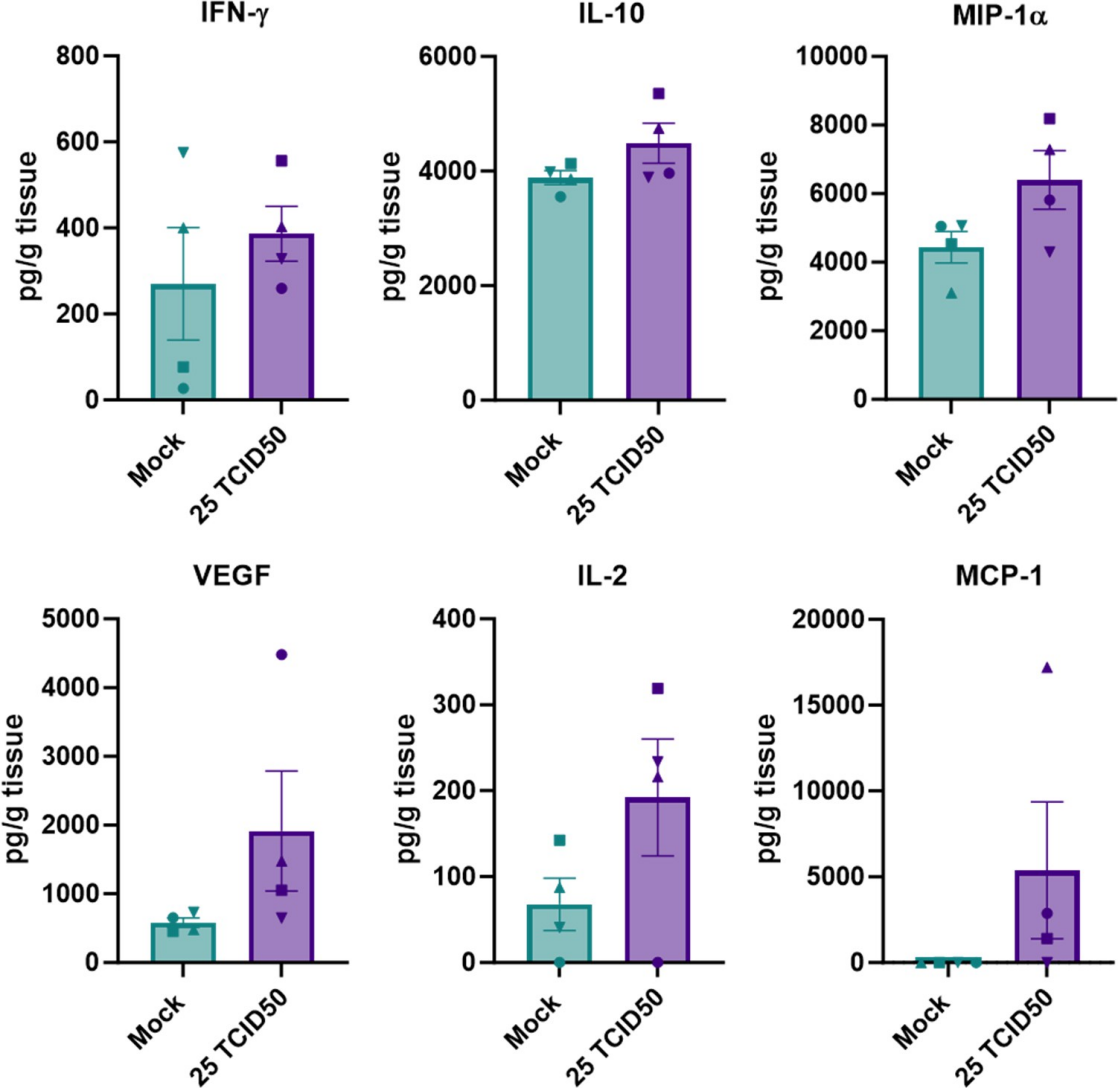
Lack of cytokine and chemokine response in brain tissue of intracranially inoculated hamsters. Brain tissues were collected at the time of euthanasia from animals inoculated intracranially with 25 TCID50 of NiV-M and mock-inoculated animals and analyzed for the presence of different cytokines and chemokines. The concentrations are plotted in pg/g tissue. Bars represent mean and error bars indicate standard error of the mean. Statistical analysis was performed using a Mann-Whitney test.

Only 6 out of 9 assayed cytokines and chemokines could be detected in brain samples (IFN-γ, IL-10, MIP-1α, VEGF, IL-2, MCP-1). Although compared to mock animals, brain samples obtained from animals inoculated with 25 TCID50 showed slightly elevated levels of IFN-γ, IL-2, IL-10, MIP-1α, and a mean difference of ~ 2000-fold in MCP-1, none of these differences were statistically significant (Fig 4).

### Histopathology in brains of intracranially inoculated animals is significantly different from classical Nipah virus lesions in the CNS

**Fig 5:**
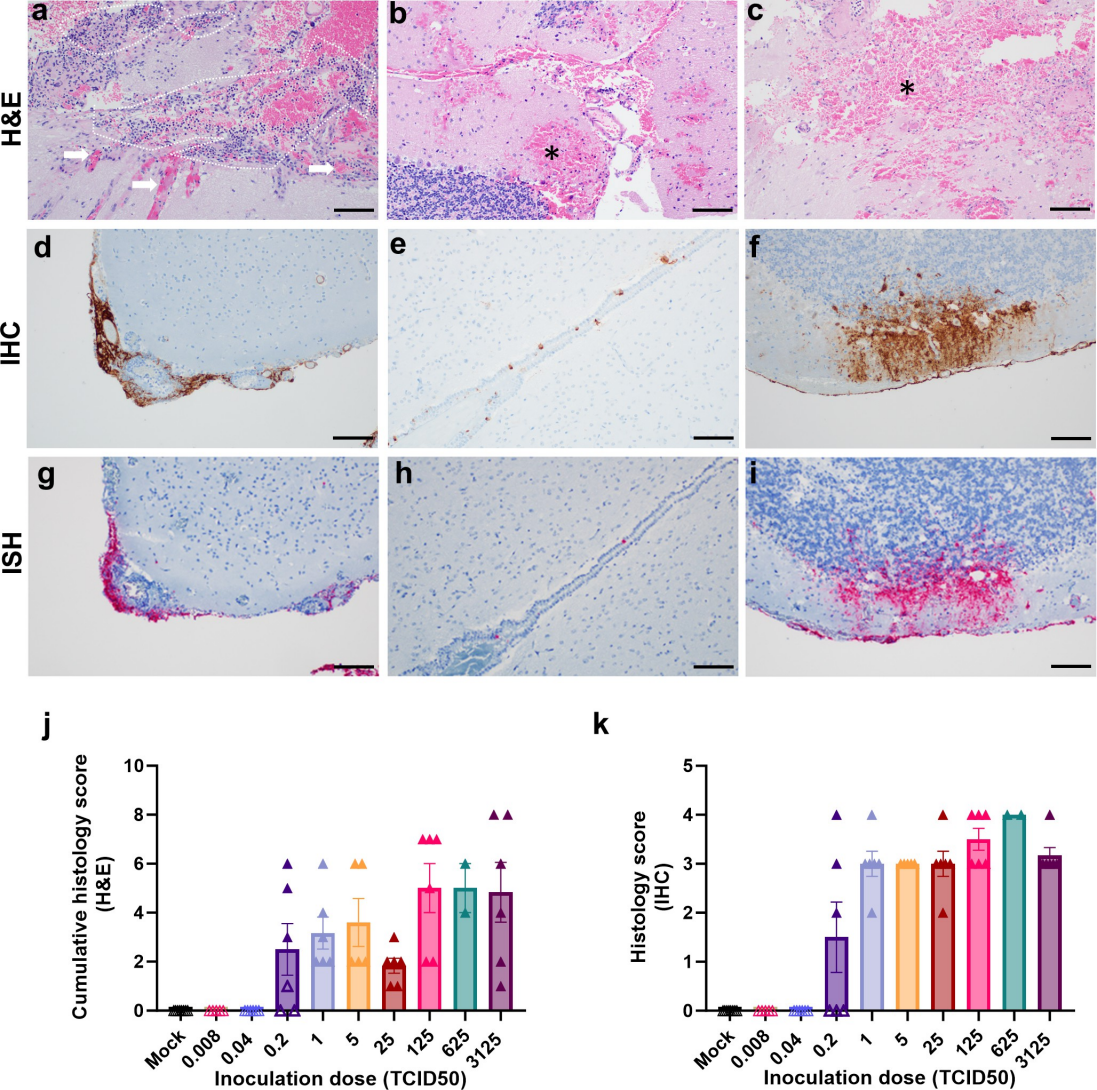
Histopathological changes are evident in the brains of all non-surviving animals. At time of euthanasia, brains were collected in formalin and sections were stained using hematoxylin and eosin (a-c), immunohistochemistry using an anti-NiV N antibody (d-f) and in situ hybridization using viral RNA probes were performed (g-i). Histological lesions included presence of occluded vessels (arrows) and inflammatory cells, primarily neutrophils (marked with dashed lines) (a), hemorrhages with necrosis (asterisk) near the injection site (b) as well as in the parenchyma (c). Immunohistochemistry and in situ hybridization revealed marked presence of viral antigen and RNA, respectively, observed within (d) and without lesions (e, f). Presence of viral antigen and RNA was detected in the leptomeninges with marked meningitis (d, g), in the ventricular lining without any surrounding inflammatory cells (e, h), and spreading from meningeal lining to cortical layer within the cerebellum (f, i). Scale bar, 100μm. Presence of histological lesions and viral antigen were scored as described in methods by a board-certified veterinary pathologist and graphed in (j) and (k). Open symbols represent animals that survived until the end of the experiment; closed symbols represent animals that reached euthanasia criteria. Bars represent mean and error bars indicate standard error of the mean.

**Fig 6:**
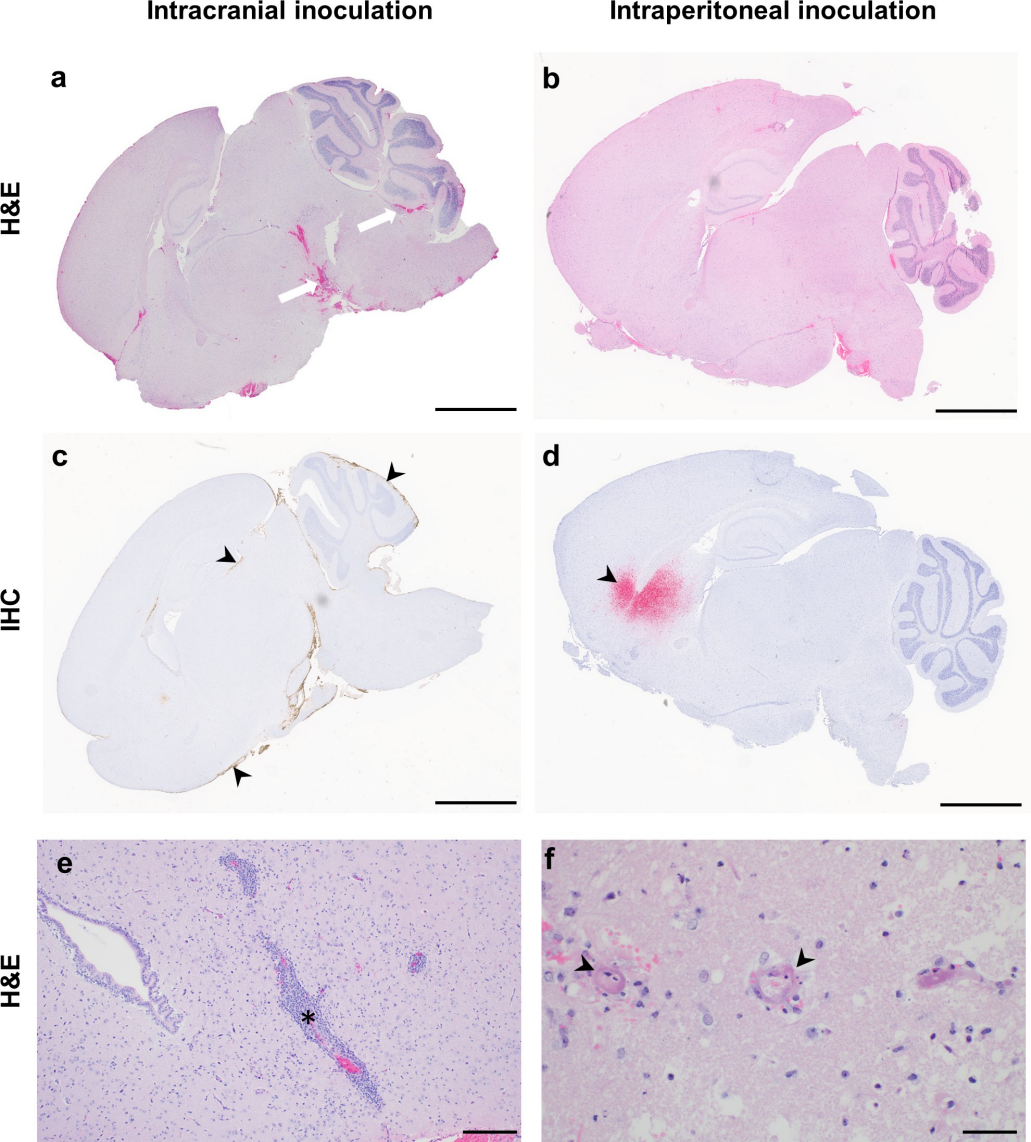
Histopathological changes in the brains of intracranially inoculated animals differ from typical NiV lesions. At time of euthanasia, brains from hamsters inoculated intracranially and intraperitoneally with NiV-M were collected in formalin. Sagittal sections were stained using hematoxylin and eosin (a, b); immunohistochemistry using an anti-NiV N antibody was performed (c, d). Representative whole brain images are shown. Hemorrhages with necrosis (white arrows) were more prevalent in brains of intracranially inoculated (a) compared to intraperitoneally inoculated animals (b). Viral antigen (brown staining, arrowhead) was observed in meningeal lining and ventricular lining of intracranially inoculated animals (c). In intraperitoneally inoculated animals, viral antigen (red staining, arrow heads) was observed within the cortex (d). Higher magnification images are shown for typical lesions such as perivascular cuffing (e, asterisks) and vasculitis (f, arrowhead) in intraperitoneally inoculated animals for comparison. Scale bars, 3mm (a-d), 200μm (e) or 50μm (f).

Histopathological changes and viral antigen were detected in the brains of all animals that reached euthanasia criteria. Major histological changes in the brains of intracranially inoculated animals included fibrinoid necrosis of small and medium-sized vessels, fibrin thrombi formation, hemorrhage, edema, and ischemic necrosis of the surrounding parenchyma (Figs 5A–5C, and S2). Large numbers of neutrophils were observed within inflamed and necrotic vessels and the surrounding parenchyma (Fig 5A). Leptomeninges were segmentally thickened by fibrin and infiltrating inflammatory cells (Fig 5D). Multifocal to regionally extensive virus antigen and viral RNA positivity was detected throughout the leptomeninges on IHC and ISH, respectively (Fig 5D and 5G). Additionally, ependymal cells lining multiple ventricles were often positive (Fig 5E and 5H). In the cerebellum, virus antigen and RNA could often be observed tracking from the leptomeninges, across the molecular layer to positive cells within the granular layer (Fig 5F and 5I). Neither lesions nor viral antigen were detected in animals who survived the intracranial challenge or mock-inoculated animals (Fig 5J and 5K). The histopathological changes observed upon intracranial inoculation (Fig 6A and S1 Table) are in stark contrast with those observed in hamsters inoculated via the intraperitoneal or intranasal routes as previously reported [12,13,18]. In animals inoculated via the intraperitoneal route that reached endpoint criteria (S3 Fig), neuropathological lesions included lymphocytic perivascular cuffing, non-suppurative encephalitic foci, and occasional vasculitis with thrombi formation (Fig 6B). The cerebellum and brain stem were the most commonly positive regions for virus antigen in hamsters that reached endpoint criteria (Fig 6C), likely due to the location of virus inoculation at the cerebellomedullary cistern. Rarely, scattered foci of viral positivity were observed throughout the parenchyma, and were not clearly associated with the meninges (Fig 6C). In contrast, viral antigen was primarily observed within the parenchyma and less often observed within the leptomeninges and ependymal cells of animals inoculated via the intraperitoneal and intranasal route (Fig 6D). Since lesions in Syrian hamsters inoculated via the intranasal or intraperitoneal route are similar to those described in humans [10] and African green monkeys [19–21], the lesions observed in intracranially inoculated hamsters do not represent NiV lesions observed during natural infection.

One of the unusual features of neuropathological lesions observed upon intracranial inoculation is the presence of large number of neutrophils. The presence of neutrophils is often caused by bacterial infection. However, no bacteria were visible in tissue slides and neutrophils were not observed in intracranially inoculated mock animals. To definitively prove that no bacterial contamination occurred during the inoculation procedure, RNA was extracted from brain tissues and 16S rRNA RT-PCR was performed. We detected a nonspecific amplified product of 650 bp (S4A Fig). Sanger sequencing showed the amplified product corresponds to a mitochondrial gene of the Syrian hamster (S4B Fig). Thus, the neutrophilic infiltrate observed in intracranially inoculated hamsters was due to NiV infection.

## Discussion

Models in which NiV-infected animals consistently display overt neurological signs are a critical tool to elucidate the neuropathogenesis of NiV and aid the development of therapeutics effective during NiV encephalitis. Here, we show that direct inoculation of NiV into the cerebellomedullary cistern results in a rapid progression to severe neurological disease. Interestingly, even a dose as low as 1 TCID50 resulted in rapid development of neurological disease requiring euthanasia. The CNS lacks a robust adaptive immune system, being protected instead by barriers such as the meninges, blood brain barrier, and blood cerebrospinal fluid barrier [22]. Direct inoculation of NiV into the cerebellomedullary cistern provides the virus an opportunity to easily breach these barriers, partially explaining the rapid development of disease. The cerebellomedullary cistern is one of the largest cerebrospinal fluid (CSF) spaces located below the cerebellum and dorsal to the medulla oblongata, and can be accessed through the foramen magnum. CSF continuously circulates throughout the CNS flowing through ventricles, subarachnoid space and cisternal spaces, including the cerebellomedullary cistern, before being reabsorbed into circulation at major absorption sites [23]. Thus, direct inoculation of virus into the cerebellomedullary cistern provides the virus immediate access to CSF circulation and, through reabsorption, potentially to blood circulation.

In hamsters, NiV could first be detected in the CNS 4 days after intranasal inoculation upon entry via olfactory neurons in the nasal cavity [12]. Hematogenous spread through virus in circulation [24] or via virus bound to leukocytes [25] requires viremia. Detectable viremia usually coincides with severe disease and thus occurs late during infection. Lastly, direct access to the CNS through breakdown of the blood brain barrier only occurs in the last stages of disease [13]. Thus, direct inoculation of the CNS reduces the time to development of neurological disease by at least 4 days. Additionally, the location of virus inoculation may have impacted disease progression. Viral antigen was mainly detected in the meningeal lining of the cerebellum and brain stem near the injection site and was often observed entering the cortical layer. Cerebellum and brain stem are neuroanatomic regions governing motor function and motor learning which may explain the most commonly observed neurological signs such as ataxia and paralysis, and thus may have led to the animals reaching euthanasia criteria soon after inoculation.

The histological lesions in the brains of intracranially inoculated hamsters were significantly different from those observed upon intranasal and intraperitoneal inoculation [12,13,18]. The most common finding after intracranial inoculation was the presence of hemorrhage with or without ischemic necrosis, a lesion that is rarely observed after intranasal or intraperitoneal inoculation. One could speculate that the hemorrhage upon intracranial inoculation is a result of intracranial pressure caused by the inoculum volume. However, this hemorrhage was not observed in animals inoculated with the same volume of DMEM, indicating that the lesion was induced by NiV replication. Neutrophils were prevalent throughout the lesions in intracranially inoculated hamsters, while rarely observed after intranasal or intraperitoneal inoculation. In fact, quantification of cells in the CNS of African green monkeys with neurological lesions showed that CD8+ and CD68+ cells are the most prevalent immune cells in these lesions [21]. Thus, the influx of neutrophils is not generally associated with NiV infection in the CNS. We excluded bacterial contamination as a cause of the observed neutrophil influx. Rather, the large number of neutrophils were likely induced by the ischemia observed in the hamsters that reached euthanasia criteria, since neutrophils are known to rapidly travel to focal areas of ischemia [26].

Taken together, our data show that although intracranial inoculation results in consistent, lethal neurological disease, the lesions observed are not consistent with those observed in NiV-infected patients [10] nor in animals infected via other routes [12,13,18,21]. Thus, although this model is of limited use in the investigation of NiV neuropathogenesis, it could potentially be used to investigate the efficacy of antivirals in the CNS. Although the window to administer antivirals is short in the model presented here, this may reflect the short time between diagnosis and fatal outcome in human patients.

## Supporting information

S1 Table Gross histologic lesion score in brains of hamsters inoculated intracranially with different doses of NiV-M. (DOCX)

S1 Fig Animals surviving intracranial Nipah virus inoculation were not infected. Serum collected from surviving animals on 14 dpi was tested for the presence of anti-Nipah virus G antibodies in ELISA. The negative control is a serum sample from a naive hamster and the positive control is serum from a hamster inoculated intranasally with Nipah virus in a previous study. Dotted line indicates the limit of detection.
(TIF)

S2 Fig Prominent histopathologic findings in the CNS of Syrian hamsters inoculated intracranially with NiV-M. A. Neutrophils (outline) within the meningeal vessels and infiltrating the leptomeninges. B. Vessel lumen filled with neutrophils, characterized by eosinophilic cytoplasm and polymorphic, segmented nuclei. C. Fibrinoid necrosis (arrowheads) of medium and small caliber vessel walls (asterisk), and associated hemorrhage (outline) into the surrounding neuropil. D. Vessel (asterisk) completely occluded by fibrin thrombi (arrowheads) and associated hemorrhage (outline) into the surrounding neuropil. H&E. Magnifications A, C, D: scale bar = 50 μm; B: scale bar = 20 μm.
(TIF)

S3 Fig Intraperitoneal inoculation of hamsters with NiV-M results in lethal disease. Groups of 8 hamsters were inoculated intraperitoneally 6800 TCID50 (100LD50) NiV-M. The percentage of animals surviving over time is shown.
(TIF)

S4 Fig Brain tissues collected from animals inoculated with Nipah virus were negative for bacterial contamination. PCR was performed for 16SrRNA sequence and a 650 bp amplified product was observed (a). Amplified product was submitted for sequencing and shown to be mitochondrial gene of Syrian hamster (X84390) (b). L denotes ladder; # denotes positive control from bacterial RNA.
(TIF)
