# Supplementary material for: Intracranial inoculation rapidly induces Nipah virus encephalitis in Syrian hamsters
Source: PLoS Negl Trop Dis. 2024 Oct 28;18(10):e0012635. doi: 10.1371/journal.pntd.0012635 (PMC11542853; doi:10.1371/journal.pntd.0012635)
Supplement: S1 Table — (DOCX) [file pntd.0012635.s001.docx]

**Table S1. Gross histologic lesion score in brains of hamsters inoculated intracranially with different doses of NiV-M.**

| **Inoculation  dose** | **Animal  ID** | **Time of euthanasia (dpi)** | **Lymphoplasmacytic meningitis** | **Lymphoplasmacytic encephalitis with gliosis** | **Malacia** | **Vasculitis, fibrin thrombi** | **Hemorrhage** | **Blood in ventricles or meninges** |
| --- | --- | --- | --- | --- | --- | --- | --- | --- |
| **Mock** | B1 | 14 | 0 | 0 | 0 | 0 | 0 | 0 |
|  | B2 | 14 | 0 | 0 | 0 | 0 | 0 | 0 |
|  | B3 | 14 | 0 | 0 | 0 | 0 | 0 | 0 |
|  | B4 | 14 | 0 | 0 | 0 | 0 | 0 | 0 |
|  | B6 | 14 | 0 | 0 | 0 | 0 | 0 | 0 |
|  | B7 | 14 | 0 | 0 | 0 | 0 | 0 | 0 |
|  | B8 | 14 | 0 | 0 | 0 | 0 | 0 | 0 |
|  | B9 | 14 | 0 | 0 | 0 | 0 | 0 | 0 |
| **0.008 TCID50** | I1 | 14 | 0 | 0 | 0 | 0 | 0 | 0 |
|  | I2 | 14 | 0 | 0 | 0 | 0 | 0 | 0 |
|  | I3 | 14 | 0 | 0 | 0 | 0 | 0 | 0 |
|  | I4 | 14 | 0 | 0 | 0 | 0 | 0 | 0 |
|  | I5 | 14 | 0 | 0 | 0 | 0 | 0 | 0 |
| **0.04 TCID50** | J1 | 14 | 0 | 0 | 0 | 0 | 0 | 0 |
|  | J2 | 14 | 0 | 0 | 0 | 0 | 0 | 0 |
|  | J3 | 14 | 0 | 0 | 0 | 0 | 0 | 0 |
|  | J4 | 14 | 0 | 0 | 0 | 0 | 0 | 0 |
|  | J5 | 14 | 0 | 0 | 0 | 0 | 0 | 0 |
|  | J6 | 14 | 0 | 0 | 0 | 0 | 0 | 0 |
| **0.2 TCID50** | K1 | 14 | 1 | 0 | 0 | 0 | 0 | 0 |
|  | K2 | 14 | 0 | 0 | 0 | 0 | 0 | 0 |
|  | K3 | 6 | 0 | 1 | 0 | 0 | 3 | 1 |
|  | K4 | 14 | 0 | 0 | 0 | 0 | 0 | 0 |
|  | K5 | 4 | 1 | 1 | 1 | 2 | 1 | 0 |
|  | K6 | 6 | 1 | 1 | 0 | 0 | 1 | 0 |
| **1 TCID50** | C1 | 4 | 0 | 0 | 0 | 2 | 3 | 1 |
|  | C2 | 3 | 0 | 0 | 0 | 1 | 1 | 1 |
|  | C3 | 4 | 0 | 1 | 1 | 2 | 0 | 0 |
|  | C4 | 4 | 0 | 0 | 0 | 1 | 0 | 1 |
|  | C5 | 4 | 0 | 0 | 0 | 1 | 1 | 0 |
|  | C6 | 4 | 1 | 0 | 0 | 1 | 0 | 0 |
| **5 TCID50** | D1 | 3 | 0 | 0 | 0 | 1 | 0 | 1 |
|  | D2 | 3 | 0 | 0 | 0 | 1 | 1 | 0 |
|  | D3 | 4 | 0 | 0 | 0 | 1 | 1 | 0 |
|  | D4 | 4 | 0 | 0 | 1 | 2 | 3 | 0 |
|  | D6 | 3 | 0 | 1 | 2 | 1 | 2 | 0 |
| **25 TCID50** | E1 | 3 | 0 | 0 | 0 | 1 | 1 | 0 |
|  | E2 | 3 | 0 | 0 | 0 | 0 | 1 | 0 |
|  | E3 | 3 | 0 | 0 | 1 | 1 | 1 | 0 |
|  | E4 | 3 | 0 | 0 | 0 | 1 | 1 | 0 |
|  | E5 | 3 | 0 | 0 | 0 | 0 | 1 | 0 |
|  | E6 | 3 | 0 | 0 | 0 | 1 | 1 | 0 |
| **125  TCID50** | F1 | 3 | 0 | 0 | 1 | 3 | 3 | 0 |
|  | F2 | 3 | 0 | 0 | 2 | 2 | 3 | 0 |
|  | F3 | 3 | 0 | 2 | 0 | 3 | 2 | 0 |
|  | F4 | 3 | 0 | 0 | 0 | 1 | 1 | 0 |
|  | F5 | 3 | 0 | 0 | 0 | 2 | 3 | 0 |
|  | F6 | 2 | 0 | 0 | 0 | 1 | 1 | 0 |
| **625 TCID50** | G3 | 3 | 0 | 0 | 0 | 2 | 2 | 0 |
|  | G5 | 3 | 0 | 0 | 0 | 2 | 3 | 1 |
| **3125 TCID50** | H1 | 2 | 0 | 0 | 1 | 3 | 2 | 0 |
|  | H2 | 2 | 0 | 0 | 0 | 1 | 1 | 0 |
|  | H3 | 2 | 0 | 0 | 2 | 3 | 3 | 0 |
|  | H4 | 2 | 0 | 0 | 0 | 0 | 1 | 0 |
|  | H5 | 2 | 0 | 0 | 1 | 4 | 3 | 0 |
|  | H6 | 2 | 0 | 0 | 0 | 2 | 2 | 0 |
