# Supplementary figures and images for: Intracranial inoculation rapidly induces Nipah virus encephalitis in Syrian hamsters
Source: PLoS Negl Trop Dis. 2024 Oct 28;18(10):e0012635. doi: 10.1371/journal.pntd.0012635 (PMC11542853; doi:10.1371/journal.pntd.0012635)

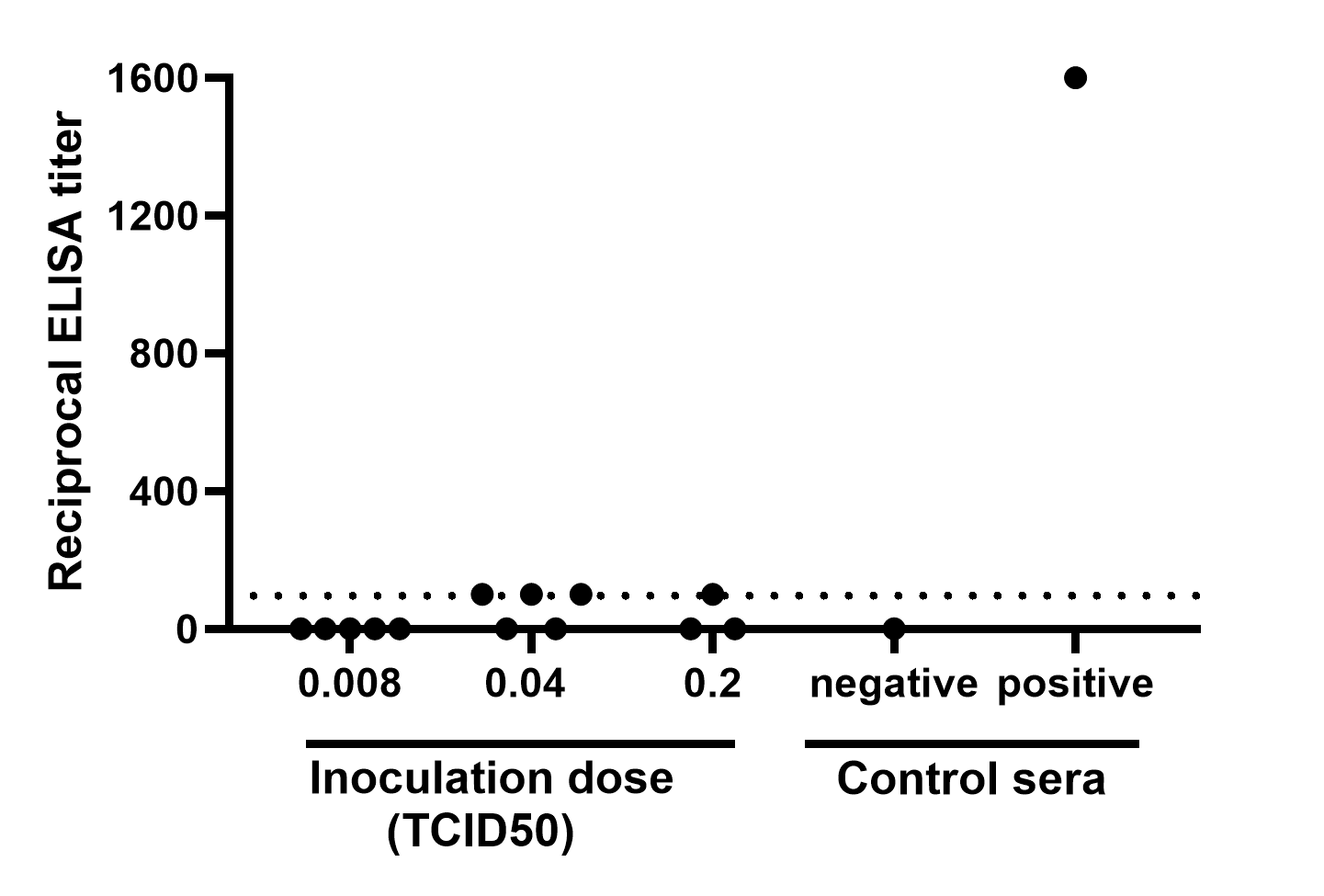

Supplement: S1 Fig — Serum collected from surviving animals on 14 dpi was tested for the presence of anti-Nipah virus G antibodies in ELISA. The negative control is a serum sample from a naive hamster and the positive control is serum from a hamster inoculated intranasally with Nipah virus in a previous study. Dotted line indicates the limit of detection. (TIF) [file pntd.0012635.s002.tif]

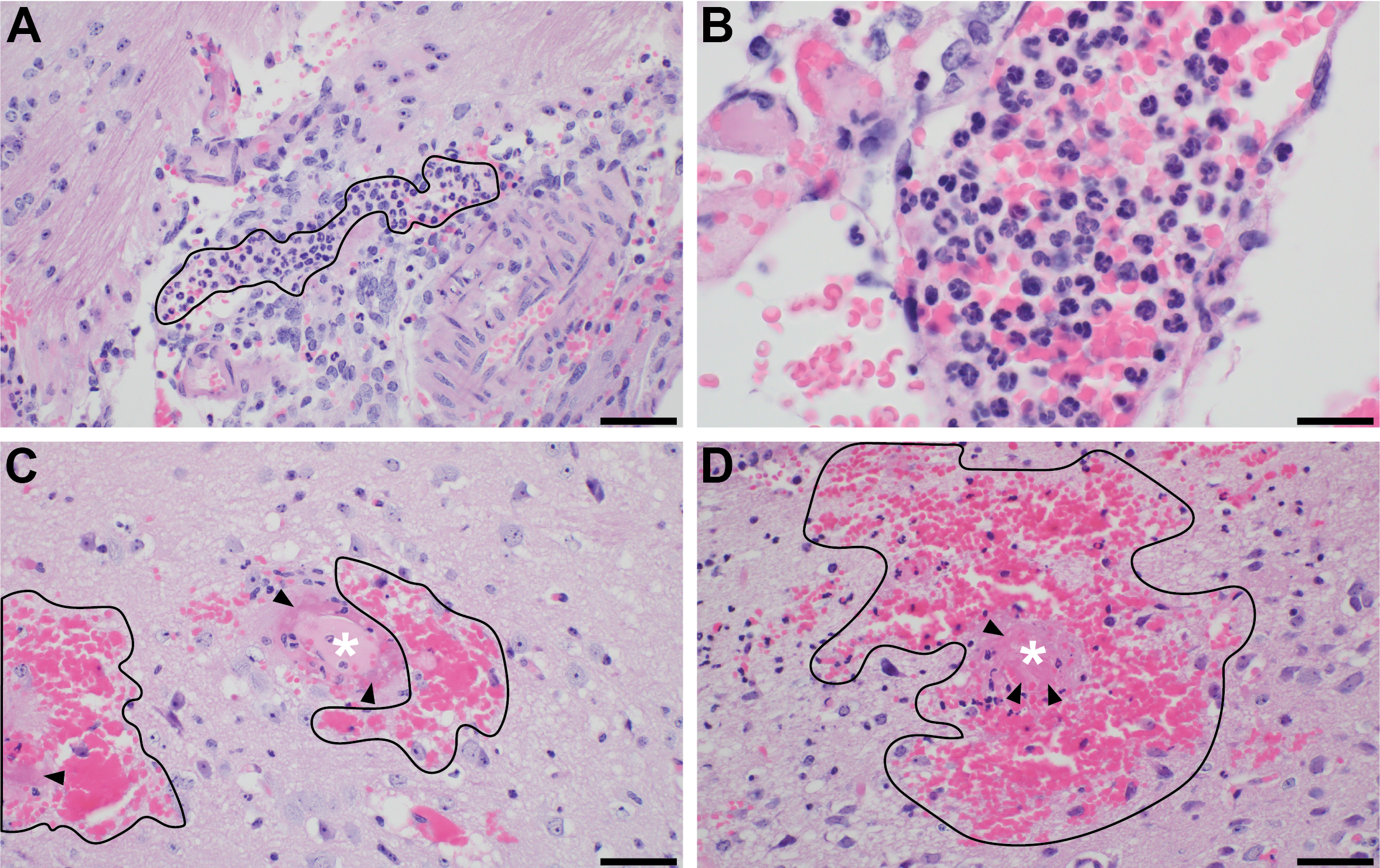

Supplement: S2 Fig — A. Neutrophils (outline) within the meningeal vessels and infiltrating the leptomeninges. B. Vessel lumen filled with neutrophils, characterized by eosinophilic cytoplasm and polymorphic, segmented nuclei. C. Fibrinoid necrosis (arrowheads) of medium and small caliber vessel walls (asterisk), and associated hemorrhage (outline) into the surrounding neuropil. D. Vessel (asterisk) completely occluded by fibrin thrombi (arrowheads) and associated hemorrhage (outline) into the surrounding neuropil. H&E. Magnifications A, C, D: scale bar = 50 μm; B: scale bar = 20 μm. (TIF) [file pntd.0012635.s003.tif]

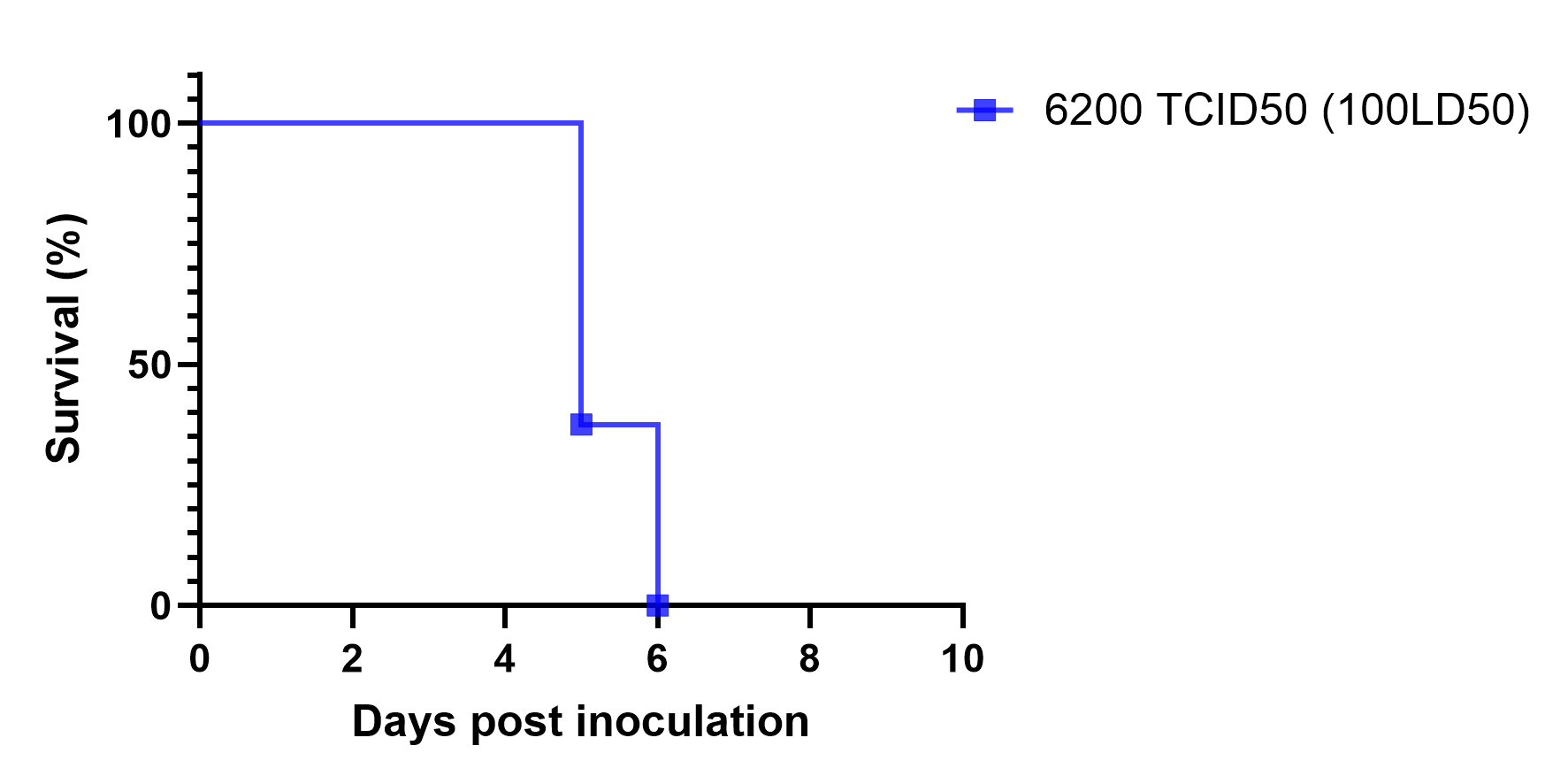

Supplement: S3 Fig — Groups of 8 hamsters were inoculated intraperitoneally 6800 TCID50 (100LD50) NiV-M. The percentage of animals surviving over time is shown. (TIF) [file pntd.0012635.s004.tif]

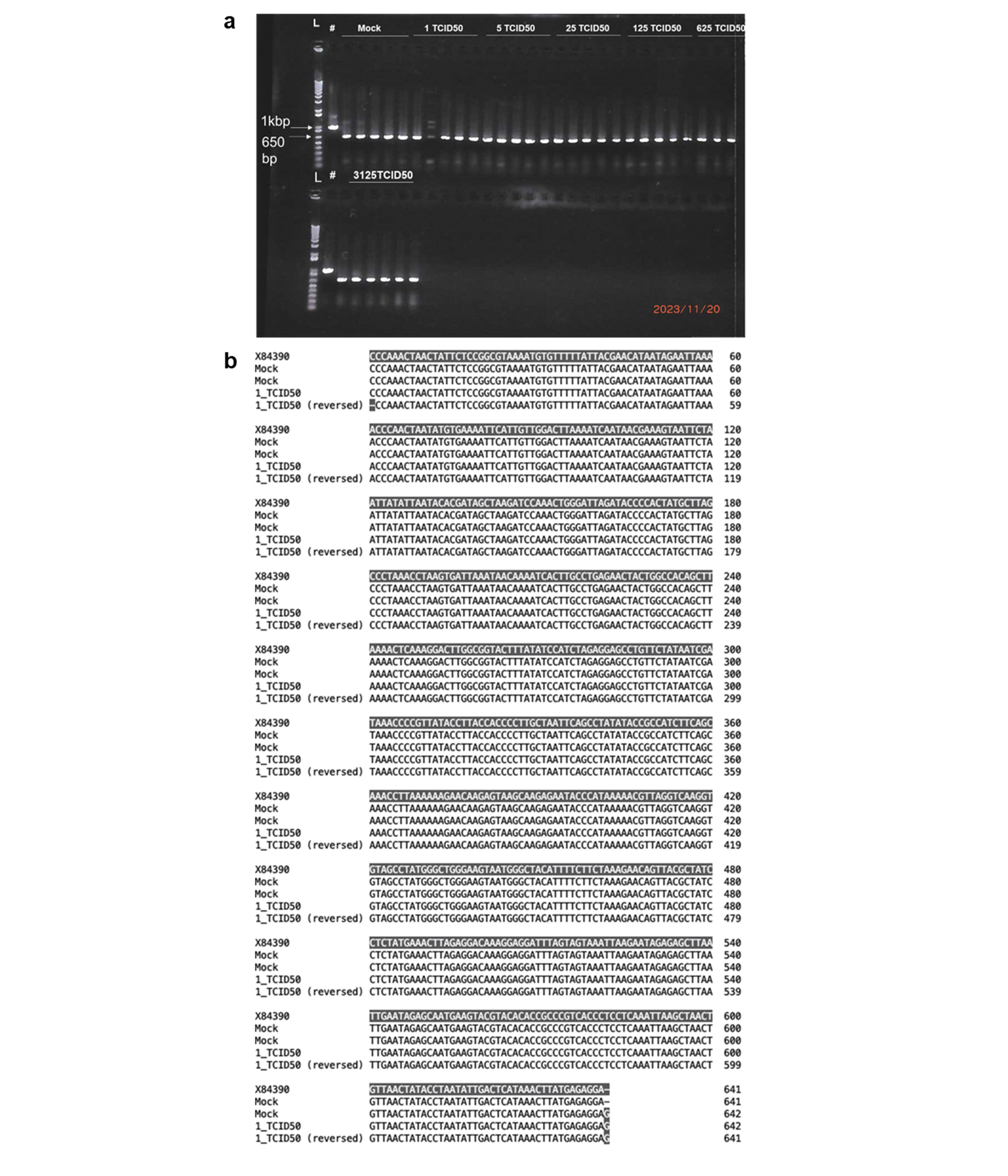

Supplement: S4 Fig — PCR was performed for 16SrRNA sequence and a 650 bp amplified product was observed (a). Amplified product was submitted for sequencing and shown to be mitochondrial gene of Syrian hamster (X84390) (b). L denotes ladder; # denotes positive control from bacterial RNA. (TIF) [file pntd.0012635.s005.tif]
